# Ideal cardiovascular health metrics and the risk of nonalcoholic fatty liver disease in Korean adults

**DOI:** 10.1186/s40885-022-00227-0

**Published:** 2023-01-15

**Authors:** Sun Young Shim, Sun Jae Jung, Seung Up Kim, Hyeon Chang Kim

**Affiliations:** 1 College of Nursing, Yonsei University, Seoul, Republic of Korea; 2 Department of Preventive Medicine, Yonsei University College of Medicine, Seoul, Republic of Korea; 3 Department of Internal Medicine, Yonsei University College of Medicine, Seoul, Republic of Korea; 4 Institute for Innovation in Digital Healthcare, Yonsei University Health System, Seoul, Republic of Korea

**Keywords:** Association, Cardiovascular diseases, Cardiovascular health, Cardiovascular health metrics, Health care quality indicators, Health status, Non-alcoholic fatty liver disease, Risk factors

## Abstract

**Background:**

The association between cardiovascular risk factors and nonalcoholic fatty liver disease (NAFLD) is well established, but whether cardiovascular health (CVH) metrics is associated with NAFLD had not been fully studied. Thus, we examined the association between CVH metrics and NAFLD in the middle-aged Korean population.

**Methods:**

We used data of 2,928 (851 men and 2,077 women) participants aged 30–64 years from the Cardiovascular and Metabolic Disease Etiology Research Center study. CVH metrics were measured using a modified version of Life’s Simple 7 by the American Heart Association. NAFLD diagnosis was based on the fatty liver index or liver-to-spleen ratio on computed tomography. A multiple logistic regression model was used to investigate the cross-sectional and longitudinal associations between CVH metrics and NAFLD.

**Results:**

In the cross-sectional analysis, the odds ratio for NAFLD was lower in participants with ideal CVH (odds ratio [OR], 0.13; 95% confidence interval [CI], 0.08–0.18), while it was higher in individuals with poor CVH (OR, 2.87; 95% CI, 2.13–3.86). Similarly, the risk of new-onset NAFLD was lower in participants with ideal CVH (OR, 0.28; 95% CI, 0.11–0.74), and higher in individuals with poor CVH (OR, 2.20; 95% CI, 0.50–9.72) in the longitudinal analysis of a subgroup.

**Conclusions:**

Ideal CVH was associated with a lower risk of NAFLD while poor CVH was associated with a higher risk of NAFLD. These findings suggest that making efforts to encourage people to manage their CVH to the ideal level may prevent and manage NAFLD.

**Supplementary Information:**

The online version contains supplementary material available at 10.1186/s40885-022-00227-0.

## Introduction

Nonalcoholic fatty liver disease (NAFLD) is an increasingly common condition and is the leading cause of chronic liver disease worldwide [1]. Over a quarter of the global population [2] and while 20%–30% of people in South Korea are estimated to have NAFLD [3, 4]. Approximately 30%–40% of people with NAFLD progress to nonalcoholic steatohepatitis, about half of whom develop hepatic fibrosis [5]. Nonalcoholic steatohepatitis patients are also at risk of developing advanced liver diseases, like cirrhosis and hepatocellular carcinoma [6]. Given the high prevalence of NAFLD and its hepatic and oncological consequences [2, 6, 7], methods should be developed to prevent and manage it. Lifestyle modifications, including increasing exercise and eating a healthy diet, remain the cornerstones of NAFLD treatment because there are no approved pharmacologic therapies [7].

The American Heart Association (AHA) designed its Life’s Simple 7 inventory including physical activity, diet, smoking, body mass index (BMI), blood pressure, fasting glucose levels, and total cholesterol to identify and promote the cardiovascular health (CVH) status [8]. Scores were generated for each of the seven elements, and scores of 0–8, 9–11, and 12–14 were categorized as poor, intermediate, and ideal, respectively [8]. While the association between individual cardiovascular risk factors and NAFLD is well established [9–11], studies on the association between CVH metrics and NAFLD were lacking. Moreover, most previous studies had examined NAFLD diagnosed by sonography; therefore, their results could not be generalized and applied to NAFLD diagnosed by other methods [12–14]. Given the public health burden of NAFLD and the limited information among the Korean population, we identified the associations between CVH metrics and NAFLD among middle-aged Koreans using a cross-sectional study. In addition, we used longitudinal analysis to confirm the temporal association between the CVH metrics and the risk of developing NAFLD.

## Methods

### Data collection and participants

This study used data from the Cardiovascular and Metabolic Diseases Etiology Research Center (CMERC) cohort study [15]. A total of 4,060 (1,426 men and 2,634 women) participants, aged 30–64 years, who had never suffered a myocardial infarction, stroke, or heart failure, had completed the CMERC baseline study examination from 2013 to 2018 were included. Participants were excluded if they were undergoing cancer treatment, had been diagnosed with cancer within the previous 2 years, were participating in clinical drug trials, or were pregnant. In the present study, the following people were also excluded: those who had liver cirrhosis or chronic hepatitis (n = 49) [5], those with a history of excessive alcohol consumption (n = 468) (defined as ≥ 30 g/day for men and ≥ 20 g/day for women [16]), and those with missing data on key variables (n = 615). Eventually, a total of 2,928 (851 men and 2,077 women) participants were included in the cross-sectional analysis.

The CMERC follow-up study of 500 participants was conducted in 2019. For the longitudinal analysis, according to the conditions for defining NAFLD, we excluded people who had been diagnosed with liver cirrhosis or chronic hepatitis (n = 16), and those with a history of excessive alcohol consumption (n = 63), at both baseline and follow-up studies [5, 16]. The remaining 421 participants were eligible for the analysis, and two cohorts were selected to determine the temporal association between CVH metrics and the risk of NAFLD. The first NAFLD cohort based on biomarkers included 308 participants, after excluding participants with NAFLD based on the fatty liver index (FLI) at baseline (n = 105) and those with missing data on key variables (n = 8). The second NAFLD cohort based on image included 335 participants, after excluding participants with NAFLD based on the liver-to-spleen ratio (LSR) at baseline (n = 72) and those with missing data on key variables (n = 14) (Fig. S1).

### Cardiovascular health metrics

We used the modified Life’s Simple 7 to measure CVH at baseline. It addresses four health behaviors including BMI, smoking, physical activity, and diet, and three health risk factors, including blood pressure, fasting glucose levels, and total cholesterol [8]. A cut-off of 23 kg/m^2^ for BMI, which has been validated for the Asian population was used in this study [17]. Smoking, physical activity, and diet were assessed through face-to-face interviews using standardized questionnaires [15]. The Korean version of the International Physical Activity Questionnaire-Short Form was used to assess physical activity [18]. Guidelines that were applicable to Asian populations for diet were used instead of the AHA’s guidelines. Diet was assessed based on the food frequency questionnaire and was categorized according to the diet quality index for Koreans (DQI-K), a tool that assesses dietary consumption and nutrition among Koreans [19, 20]. The DQI-K contains components of daily protein intake, the percentage of energy obtained from fat, the percentage of energy obtained from saturated fat, daily cholesterol intake, daily whole-grain intake, daily vegetable intake, daily fruit intake, and daily sodium intake. Each component was scored according to a cut-off, with a total score ranged from 0 to 9 (Table S1). Blood pressure was measured three times on the right arm, with the participants in a seated posture. The mean of the last two measurements was used in the analysis. Overnight fasting blood samples taken from the antecubital vein were obtained in the morning by a trained researcher.

The total CVH metrics scores were calculated as the sum of the scores for each component for a possible range of 0–14 (Table S2). Scores 0–7 were considered as poor, 8–11 as intermediate, and 12–14 as ideal CVH metrics in the cross-sectional analysis [21]. The diet component was not measured in the longitudinal analysis because there was no baseline diet information for more than half of the longitudinal study participants. Thus, the total scores of 0–6 were classified as poor, 7–9 as intermediate, and 10–12 as ideal CVH metrics in the longitudinal analysis.

### Nonalcoholic fatty liver disease

NAFLD was diagnosed based on biomarkers as well as imaging studies and the details were described in the Table 1. First, the FLI, one of the best-validated steatosis scores that has also been validated in the Korean population [22], was used to diagnose NAFLD [23]. Second, the LSR calculated by measuring the lumbar spine L1 level and L4 levels using quantitative computed tomography was used to diagnose NAFLD [15, 17]. All images were analyzed using QCT PRO software (Mindways Software, Austin, TX, USA) and the CTXA Hip Exam Analysis protocol (Mindways Software) [15].

**Table 1:** Diagnosis of nonalcoholic fatty liver disease in the study

Variable	Index	Formula	Cut-point
Biomarker	Fatty liver index	$1/((1+exp(-x)))\times 100,$	≥ 30
		$$\begin{array}{c}x= 0.953 \times {log}_{e} \left(triglycerides\right)+ 0.139 \times BMI + 0.718 \times {log}_{e} \left(\gamma -glutamyl-transferase\right)\\ + 0.053 \times \left(waist\ circumference\right)- 15\end{array}$$	
	NAFLD liver fat score^a)^	$$\begin{array}{c}-2.89 + 1.18 \times metabolic\ syndrome \left(yes =1, no =0\right)+ 0.45\times diabetes \left(yes =2, no =0\right)\\ + 0.15 \times (fasting\ insulin) + 0.04 \times AST + 0.94 \times \frac{AST}{ALT}\end{array}$$	> –0.64
	Hepatic steatosis index ^a)^	$8\times \frac{ALT}{AST}+BMI \left(+2, if\ diabetes; +2, if female\right)$	> 36
Image using quantitative computed tomography	Liver-to-spleen ratio	$The\ mean\ value\ of\ liver\ ROI\ (HU)^\text{b)}/\ the\ mean\ value\ of\ spleen\ ROI\ (HU)$	< 1.0

*BMI* Body mass index, *NAFLD* Nonalcoholic fatty liver disease, *AST* Aspartate aminotransferase, *ALT* Alanine aminotransferase, *ROI* Region of interest, *HU* Hounsfield unit

^a)^Used for the sensitivity analyses

^b)^The values measured from one ROI at baseline examination and the average of the values measured from three ROIs at follow-up study were respectively used

### Covariates

We adjusted for age, sex, annual household income, education level, high-density lipoprotein cholesterol, triglycerides, alanine aminotransferase, homeostatic model assessment for insulin resistance (HOMA-IR), and alcohol intake in analyses based on previous studies [12, 14, 24, 25]. All covariates were continuous variables except for sex and education level. Education level was categorized as either primary or below, secondary, or post-secondary [12]. HOMA-IR was calculated by multiplying fasting insulin levels (U/mL) by fasting glucose levels (mg/dL) and dividing the result by 405 [26].

### Statistical analysis

Baseline differences between the categories of CVH metrics were compared using the Kruskal–Wallis test, analysis of variance for continuous variables, and the chi-square test for categorical variables. Differences between the general characteristics of the included people and excluded people in this study were evaluated using the 2-sample t-test or the chi-square test. For the cross-sectional analysis, multiple logistic regression models adjusted for the covariates were used to estimate the association between CVH metrics and NAFLD at baseline. These analyses included both categorical and continuous CVH metrics scores. We divided the CVH metrics into behavioral and biomarker components [27] and confirmed that these two groups were associated with NAFLD via a multiple logistic model. For longitudinal analysis, we conducted a multiple logistic regression model to confirm a temporal association between CVH metrics at baseline and the risk of developing NAFLD after 5 years while adjusting for the same covariates used in the cross-sectional analysis.

Sensitivity analyses were conducted as follows. First, different biomarker-based prediction models such as NAFLD liver fat score (NAFLD-LFS) [22, 28] and hepatic steatosis index (HSI) [29], and the combination of biomarker-based prediction models and LSR (FLI plus LSR, NAFLD-LFS plus LSR, HSI plus LSR) were used to confirm the association between CVH metrics and NAFLD. Second, different tools for measuring CVH, such as the Cardiovascular Health in Ambulatory Care Research Team (CANHEART) index [30] and the 10-year risk for atherosclerotic cardiovascular disease (ASCVD) [31] were used to replicate our main results. The CANHEART health index was developed to comprehensively examine the CVH of Canadians [30]. The components of the CANHEART health index are the same as those of Life’s Simple 7, except that it does not contain a metric for cholesterol. However, it has different cut-offs and method for measuring each component [30]. The 10-year risk for ASCVD was calculated using a Korean ASCVD prediction model for participants aged ≥ 40 years [31].

Odds ratios (ORs) with 95% confidence intervals (CIs) or *P*-values were reported as measures of effect size. All statistical tests were performed using SAS ver. 9.4 (SAS Institute Inc., Cary, NC, USA).

### Ethics statement

All participants provided a written informed consent to participate in the CMERC baseline and follow-up studies. The protocol of this was approved by the Institutional Review Board of Severance Hospital at Yonsei University College of Medicine (IRB No: 4–2013-0661). All procedures in this work complied with the Helsinki Declaration of 1975, as revised in 2008.

## Results

### Baseline characteristics of the study population

The participants’ mean age was 51.8 years and 851 of them (29.1%) were men (Table 2). FLI-based NAFLD was observed in 30.0% of the participants, and image-based NAFLD was observed in 16.2%. The proportion of people with ideal CVH was 17.6%, 10.7%, and 20.4% for all participants, men, and women, respectively (Fig. S2). Newly two-thirds of the sample had intermediate CVH. As the CVH metric level increased from poor to ideal, the blood pressure, BMI, fasting glucose, total cholesterol, and proportion of smokers decreased (P ≤ 0.001). Education level and household income increased as the CVH category level increased, but it was not significant (P = 0.084, P = 0.268, respectively). The proportion of hypertension, diabetes and NAFLD decreased as the CVH category level increased (all P ≤ 0.001). Compared to the excluded people, the study participants were older, had a higher proportion of women, and had lower blood pressure, BMI, total energy intake and fasting glucose level (Table S3).

**Table 2:** Baseline characteristics of study participants according to category of cardiovascular health metrics

Characteristic	Total sample	Cardiovascular health metrics			*P*-value^a)^
		Poor	Intermediate	Ideal	
Age (yr)	51.8 ± 9.3	51.4 ± 9.5	52.3 ± 9.1	50.2 ± 9.8	< 0.001
Sex					< 0.001
Male	851 (29.1)	253 (51.2)	507 (26.4)	91 (17.7)	
Female	2,077 (70.9)	241 (48.8)	1,413 (73.6)	423 (82.3)	
Systolic BP (mmHg)	117.9 ± 14.9	128.9 ± 15.5	117.4 ± 14.0	108.9 ± 10.4	< 0.001
Diastolic BP (mmHg)	75.5 ± 9.7	82.5 ± 10.4	75.2 ± 9.1	70.2 ± 7.4	< 0.001
Body mass index (kg/m^2^)	23.7 ± 3.0	26.5 ± 2.9	23.6 ± 2.8	21.5 ± 1.7	< 0.001
Fasting glucose (mg/dL)	88.0 [82–95]	101.0 [86–108]	88.0 [82–94]	85.0 [80–90]	< 0.001
HOMA-IR	2.1 ± 1.1	2.9 ± 1.6	2.0 ± 0.9	1.6 ± 0.6	< 0.001
Total cholesterol (mg/dL)	199.5 ± 35.5	211.2 ± 41.4	200.1 ± 34.8	186.4 ± 26.9	< 0.001
HDL-C (mg/dL)	57.8 ± 14.5	52.4 ± 12.4	58.3 ± 14.6	61.2 ± 14.5	< 0.001
Triglycerides (mg/dL)	104.0 [75–146]	138.5 [102–197]	102.5 [75–143]	84.0 [62–112]	< 0.001
Total energy intakes (kcal/day)	2,148.0 ± 758.2	2,359.4 ± 810.2	2,137.9 ± 747.8	1,982.5 ± 697.7	< 0.001
Current smoker	272 (9.3)	147 (29.8)	122 (6.4)	3 (0.6)	< 0.001
Alcohol intake^b)^	369 (12.6)	112 (22.7)	218 (11.4)	39 (7.6)	< 0.001
High education^c)^	1,395 (47.6)	225 (45.6)	910 (47.4)	260 (50.6)	0.084
High income^d)^	1,663 (56.8)	265 (53.6)	1,098 (57.2)	300 (58.4)	0.268
Hypertension	711 (24.3)	250 (50.6)	439 (22.9)	22 (4.3)	< 0.001
Diabetes	254 (8.7)	125 (25.3)	124 (6.5)	5 (1.0)	< 0.001
ALT (U/L)	24.8 ± 17.2	33.8 ± 25.4	23.6 ± 13.4	20.7 ± 16.9	< 0.001
γ-GTP (U/L)	19 [14–29]	29 [19–47]	19 [14–28]	15 [12–20]	< 0.001
NAFLD by FLI	877 (30.0)	344 (69.6)	512 (26.7)	21 (4.1)	< 0.001
NAFLD by LSR	473 (16.2)	187 (37.9)	261 (13.6)	25 (4.9)	< 0.001

Values are presented as mean ± standard deviation, median [interquartile range], or number (%)

*BP* Blood pressure, *HDL-C* High-density lipoprotein cholesterol, *ALT* Alanine aminotransferase, *γ-GTP* γ-glutamyl transpeptidase, *NAFLD* Nonalcoholic fatty liver disease, *FLI* Fatty liver index, *LSR* Liver-to-spleen ratio

^a)^The *P*-values were calculated through analysis of variance, or Kruskal–Wallis test, or chi-square test where appropriate

^b)^ ≥ 10 g of alcohol per day

^c)^College graduate or above

^d)^Annual household income ≥ 50,800$

### Cross-sectional association between CVH metrics and NAFLD

Table 3 shows the associations between CVH metrics and NAFLD as determined by the multiple logistic regression model that adjusted for covariates. A total of 2,928 participants were included in the cross-sectional analysis. When compared to the intermediate CVH group, the ideal CVH group had lower odds of having FLI-based NAFLD (OR, 2.87; 95% CI, 2.13–3.86) and image-based NAFLD (OR, 1.63; 95% CI, 1.24–2.14), while the poor CVH group had higher odds for having FLI-based NAFLD (OR, 0.13; 95% CI, 0.07–0.23) and image-based NAFLD (OR, 0.55; 95% CI, 0.35–0.86). These independent associations were similarly found in both men and women (Table S4). As the continuous score of CVH metrics increased by 1 point, the adjusted ORs for NAFLD diagnosed by FLI and LSR was 0.63 (95% CI, 0.59–0.68) and 0.83 (95% CI, 0.78–0.89), respectively (Table 3). The continuous scores of the four behavioral components and that of three biomarker components were also associated with a lower risk of NAFLD. Among biomarker components, ideal blood pressure was independently associated with a lower risk of NAFLD (OR, 0.65; 95% CI, 0.50–0.84, data not shown).

**Table 3:** Association between cardiovascular health metrics and the risk of nonalcoholic fatty liver disease

Cardiovascular health metrics	No. of people	NAFLD by fatty liver index			NAFLD by liver-to-spleen ratio		
		No. (%) of people with NAFLD	Unadjusted OR (95% CI)	Adjusted OR (95% CI)	No. (%) of people with NAFLD	Unadjusted OR (95% CI)	Adjusted OR (95% CI)
Category (score)							
Poor (0–7)	494	344 (69.6)	6.31 (5.08–7.83)	2.87 (2.13–3.86)	187 (37.9)	3.87 (3.10–4.84)	1.63 (1.24–2.14)
Intermediate (8–11)	1,920	512 (26.7)	1.00	1.00	261 (13.6)	1.00	1.00
Ideal (12–14)	514	21 (4.1)	0.12 (0.08–0.18)	0.13 (0.07–0.23)	25 (4.9)	0.33 (0.21–0.50)	0.55 (0.35–0.86)
Continuous score, per 1.0							
Overall	2,928	877 (30.0)	0.52 (0.49–0.55)	0.63 (0.59–0.68)	473 (16.2)	0.66 (0.62–0.69)	0.83 (0.78–0.89)
Behavioral components	2,928	877 (30.0)	0.52 (0.49–0.56)	0.55 (0.50–0.60)	473 (16.2)	0.68 (0.64–0.73)	0.86 (0.79–0.93)
Biomarker components	2,928	877 (30.0)	0.52 (0.48–0.56)	0.77 (0.69–0.87)	473 (16.2)	0.56 (0.52–0.61)	0.77 (0.69–0.86)

Adjusted for age, sex, high-density lipoprotein cholesterol, triglycerides, HOMA-IR, alanine aminotransferase, income, education level and alcohol intake

*NAFLD* Nonalcoholic fatty liver disease, *OR* Odds ratio, *CI* Confidence interval, *HOMA-IR* Homeostatic model assessment for insulin resistance

### Longitudinal association between CVH metrics and NAFLD

We investigated the temporal association between CVH metrics at the baseline examination and new-onset NAFLD at the follow-up examination after 5 years (Table 4). Participants with NAFLD at baseline, and those with missing data on key variables were also excluded. A total of 308 and 335 participants were included in the NAFLD cohort based on biomarkers and the NAFLD cohort based on image, respectively. At the follow-up examination, 28 and 20 cases of FLI-based NAFLD and LSR-based NAFLD, respectively, were newly diagnosed. When NAFLD was diagnosed by FLI, ideal CVH was associated with a lower incidence of NAFLD (OR, 0.28; 95% CI, 0.11–0.74), whereas poor CVH was associated with a higher incidence of NAFLD (OR, 2.20; 95% CI, 0.50–9.72). As the continuous score of CVH metrics increased by 1 point at baseline, the OR for the risk of developing new-onset NAFLD was 0.71 (95% CI, 0.55–0.92). These results were consistent with those of the cross-sectional analysis. Meanwhile, when NAFLD was diagnosed by LSR, ideal CVH was not associated with NAFLD (OR, 3.12; 95% CI, 0.87–11.20) and poor CVH was associated with a higher incidence of NAFLD (OR, 4.83; 95% CI, 1.14–20.43). Analysis of the association between continuous CVH metrics scores at baseline and the risk of developing NAFLD diagnosed by LSR had a significantly decreased unadjusted OR (OR, 0.75; 95% CI, 0.59–0.96). However, the strength of this association was attenuated after adjusting for potential confounders (OR, 0.95; 95% CI, 0.71–1.27).

**Table 4:** Association between cardiovascular health metrics at baseline examination and the risk of developing nonalcoholic fatty liver disease after 5 years

Cardiovascular health metrics	NAFLD diagnosed by fatty liver index				NAFLD diagnosed by liver-to-spleen ratio			
	No. of people	No. (%) of people with NAFLD	Unadjusted OR (95% CI)	Adjusted OR (95% CI)	No. of people	No. (%) of people with NAFLD	Unadjusted OR (95% CI)	Adjusted OR (95% CI)
Category (score)								
Poor (0–6)	16	3 (18.8)	1.58 (0.41–6.08)	2.20 (0.50–9.72)	27	6 (22.2)	7.43 (2.19–25.16)	4.83 (1.14–20.43)
Intermediate (7–9)	141	18 (12.8)	1.00	1.00	162	6 (3.7)	1.00	1.00
Ideal (10–12)	151	7 (4.6)	0.33 (0.13–0.82)	0.28 (0.11–0.74)	146	8 (5.5)	1.51 (0.51–4.45)	3.12 (0.87–11.20)
Continuous score								
Per 1	308	28 (9.1)	0.76 (0.61–0.95)	0.71 (0.55–0.92)	335	20 (6.0)	0.75 (0.59–0.96)	0.95 (0.71–1.27)

Adjusted for age, sex, high-density lipoprotein cholesterol, triglycerides, HOMA-IR, alanine aminotransferase, income, education level and alcohol intake

*NAFLD* Nonalcoholic fatty liver disease, *OR* Odds ratio, *CI* Confidence interval, *HOMA-IR* Homeostatic model assessment for insulin resistanc

### Sensitivity analysis

Sensitivity analysis was conducted by first, investigating the association between CVH metrics and NAFLD diagnosed by NAFLD-LFS, HSI, FLI plus LSR, NAFLD-LFS plus LSR, and HSI plus LSR. Each of these analyses produced similar results to the results of diagnosing NAFLD with FLI and LSR (Table S5). Second, we used the CANHEART index and 10-year risk for ASCVD to measure CVH and confirm the association between CVH and NAFLD. When CVH was measured with the CANHEART health index, ideal CVH was associated with a decreased prevalence of NAFLD, and poor CVH was associated with an increased prevalence of NAFLD, which was consistent with those measured using Life’s Simple 7. The group with a 10-year risk for ASCVD of ≥ 5% was also associated with a higher prevalence of NAFLD, which was consistent with the finding that poor CVH was associated with a higher prevalence of NAFLD (Table S6).

## Discussion

We examined the association between CVH metrics and NAFLD among middle-aged adults in Korea. Ideal CVH was associated with a lower prevalence of NAFLD while poor CVH was associated with a higher prevalence of NAFLD in a cross-sectional analysis. These associations were observed in both, the FLI-diagnosed NAFLD and the LSR-diagnosed NAFLD cases. The longitudinal analysis showed that ideal CVH was associated with a lower risk of developing new-onset NAFLD, while poor ideal CVH was associated with a higher risk of developing NAFLD than intermediate CVH. However, this temporal association was attenuated when NAFLD was diagnosed using the LSR. This result was likely because the liver's mean Hounsfield unit (HU), which was used to calculate LSR, was measured in different ways in the baseline and follow-up examinations.

Only a few studies have reported the association between CVH metrics measured using Life’s Simple 7 and NAFLD. Our results were consistent with those of previous studies. A cross-sectional study of 10,511 participants in northern China reported that NAFLD diagnosed by abdominal ultrasonography was associated with both the number of ideal CVH components and the total CVH metrics scores [12]. Another study in China using prospective cohort data showed that the incidence of NAFLD decreased as the number of ideal CVH metrics increased over a 6-year follow-up period [13]. However, both studies were limited by the fact that they did not measure diet or physical activity according to the AHA guidelines [12, 13]. A cross-sectional study was conducted with 3,901 participants of various ethnicities and found that ideal CVH was associated with a lower prevalence of NAFLD as diagnosed by cardiac computed tomography [32]. Another cross-sectional study of 23,227 adults in the United States using National Health and Nutrition Examination Survey data reported that individuals with an ideal CVH had a 12% lower chance of having NAFLD diagnosed by FLI than those with poor CVH [24].

Several tools for measuring CVH have been developed in recent decades. For example, the Framingham risk score is calculated using the Framingham multivariate equation, which was designed to estimate the 10-year risk of developing coronary heart disease [33]. The American College of Cardiology and AHA modified the Framingham risk score to develop pooled cohort equations to estimate the 10-year risk of a first ASCVD event [34]. The Korean risk prediction model was designed by modifying these equations using Korean heart study data [31]. In the United Kingdom, the cardiovascular disease risk algorithm was derived to identify people at high risk of cardiovascular disease [35]. However, because these equations are complex, it is difficult for the general public to use without a dedicated program. Life's Simple 7 does not require complex calculations and shows people simple steps that they can take to improve CVH to prevent hypertension, cardiovascular disease and many other chronic disorders. Specific and challenging goals are more likely to result in successfully changing health behaviors than vague goals [36]. Self-monitoring goal-related progress is crucial to achieving goals because it provides individuals with the information they need to adjust their strategies or levels of effort [36, 37]. In this regard, Life's Simple 7 is an effective tool for setting and monitoring goals to prevent both cardiovascular disease and NAFLD because the cut-off for each component is clearly and simply defined. Life's Simple 7 can be assessed and interpreted at the individual or population level [8]. Therefore, the management of NAFLD with Life's Simple 7 is useful for evaluating population health, identifying groups at high risk of developing diseases, and identifying directions for improving population-wide health. Eventually, monitoring the population using Life Simple 7 can contribute to improving not only cardiovascular health but also preventing metabolic diseases.

Our results should be interpreted with caution when applying them to other populations because of the differences between participants and those who were excluded. The study participants were healthier than those who were excluded because we excluded those who consumed a lot of alcohol and had liver disease. Thus, it could be predicted that those excluded were likely to have more accumulated liver fat and worse CVH than the study participants. Therefore, the effect size and strength of our results may be attenuated by excluding these individuals.

This study has some limitations to be discussed. First, the diagnosis of NAFLD using the FLI or LSR is not as accurate as that using liver biopsies. Nonetheless, FLI has been utilized in many epidemiologic studies as a tool with high accuracy in validity studies [22, 23]. LSR is also an effective tool for diagnosing NAFLD, which has been well correlated with the degree of steatosis shown in histopathology [38]. The second limitation concerns measurement errors in behavioral components such as smoking, physical activity, and diet [39–42]. Self-reported smoking responses underestimate the smoking rates among Korean women, compared to smoking assessed by measuring the urinary cotinine levels, which may have resulted in a bias in this study [39]. However, these random misclassification biases would skew the results towards a null association. Furthermore, we confirmed that the high 10-year risk for ASCVD, calculated using mainly biomarker variables instead of self-reported variables [31], was also associated with a higher prevalence of NAFLD. Third, we could not confirm the association between CVH metrics and NAFLD according to fibrotic burden because the number of people with NAFLD and severe fibrosis among our participants was not large enough for an analysis. Further studies on the relationship between CVH metrics and NAFLD according to the fibrotic burden are needed. Fourth, although we included risk factors for NAFLD in the adjusted model, there may be residual confounding such as inflammation markers and comorbidities that we did not control for. Lastly, the participants were limited to middle-aged Korean adults, so the results may not be widely generalizable.

## Conclusions

In conclusion, our study evaluated the association between CVH metrics and NAFLD to provide information on the prevention and management of NAFLD among Korean adults. We found strong associations between ideal CVH and a low risk of NAFLD, and poor CVH and a high risk of NAFLD. These results were consistent with those of the sensitivity analyses. This finding suggests that making efforts to encourage people to manage their CVH to the ideal level may prevent and manage not only cardiovascular disease but also NAFLD.

## Supplementary Information

**Additional file 1: Fig. S1.** Flowchart of study population selection for longitudinal analysis. **Fig. S2.** Distribution of cardiovascular health metrics. **Table S1.** Components and cut-points in the Diet Quality Index for Koreans (DQI-K). **Table S2.** Definition of cardiovascular health metrics. **Table S3. **Baseline characteristics of participants and those excluded from the study. **Table S4.** Association between cardiovascular metrics categories and the risk of nonalcoholic fatty liver disease according to sex. **Table S5.** Association between cardiovascular metrics and nonalcoholic fatty liver disease. **Table S6.** Association between the Cardiovascular Health in Ambulatory Care Research Team (CANHEART) health index, 10-year risk for atherosclerotic cardiovascular disease and the risk of non-alcoholic fatty liver disease.

## Data Availability

The datasets used and/or analyzed during the current study are available from the corresponding author on reasonable request.

## Abbreviations

NAFLD: Nonalcoholic fatty liver disease
AHA: American Heart Association
BMI: Body mass index
CVH: Cardiovascular health
CMERC: Cardiovascular and Metabolic Diseases Etiology Research Center
FLI: Fatty liver index
LSR: Liver-to-spleen ratio
DQI-K: Diet quality index for Koreans
HOMA-IR: Homeostatic model assessment for insulin resistance
NAFLD-LFS: NAFLD liver fat score
HSI: Hepatic steatosis index
CANHEART: Cardiovascular Health in Ambulatory Care Research Team
ASCVD: Atherosclerotic cardiovascular disease
OR: Odds ratio
CI: Confidence interval
HU: Hounsfield unit
