# Supplementary material for: Ideal cardiovascular health metrics and the risk of nonalcoholic fatty liver disease in Korean adults
Source: Clin Hypertens. 2023 Jan 15;29:3. doi: 10.1186/s40885-022-00227-0 (PMC9840828; doi:10.1186/s40885-022-00227-0)
Supplement: Supplementary file 1 — Additional file 1: Fig. S1. Flowchart of study population selection for longitudinal analysis. Fig. S2. Distribution of cardiovascular health metrics. Table S1. Components and cut-points in the Diet Quality Index for Koreans (DQI-K). Table S2. Definition of cardiovascular health metrics. Table S3. Baseline characteristics of participants and those excluded from the study. Table S4. Association between cardiovascular metrics categories and the risk of nonalcoholic fatty liver disease according to sex. Table S5. Association between cardiovascular metrics and nonalcoholic fatty liver disease. Table S6. Association between the Cardiovascular Health in Ambulatory Care Research Team (CANHEART) health index, 10-year risk for atherosclerotic cardiovascular disease and the risk of non-alcoholic fatty liver disease. [file 40885_2022_227_MOESM1_ESM.docx]

v

335 Cohort of non-alcoholic fatty liver disease based on image

Follow-up study population (2019), n = 500

308 Cohort of non-alcoholic fatty liver disease based on biomarker

Exclusion

- Excessive alcohol drinker: 63 (baseline, 49; follow-up, 14)
- Liver disease (cirrhosis or chronic hepatitis): 16 (baseline, 15; follow-up, 1)

421 Participants were eligible in the analysis

Exclusion

- 105 Non-alcoholic fatty liver disease was diagnosed by fatty liver index at baseline

- 8 Key variable missing

Exclusion

- 72 Non-alcoholic fatty liver disease was diagnosed by quantitative computed tomography at baseline

- 14 Key variable missing

**Fig. S1.** Flowchart of study population selection for longitudinal analysis.


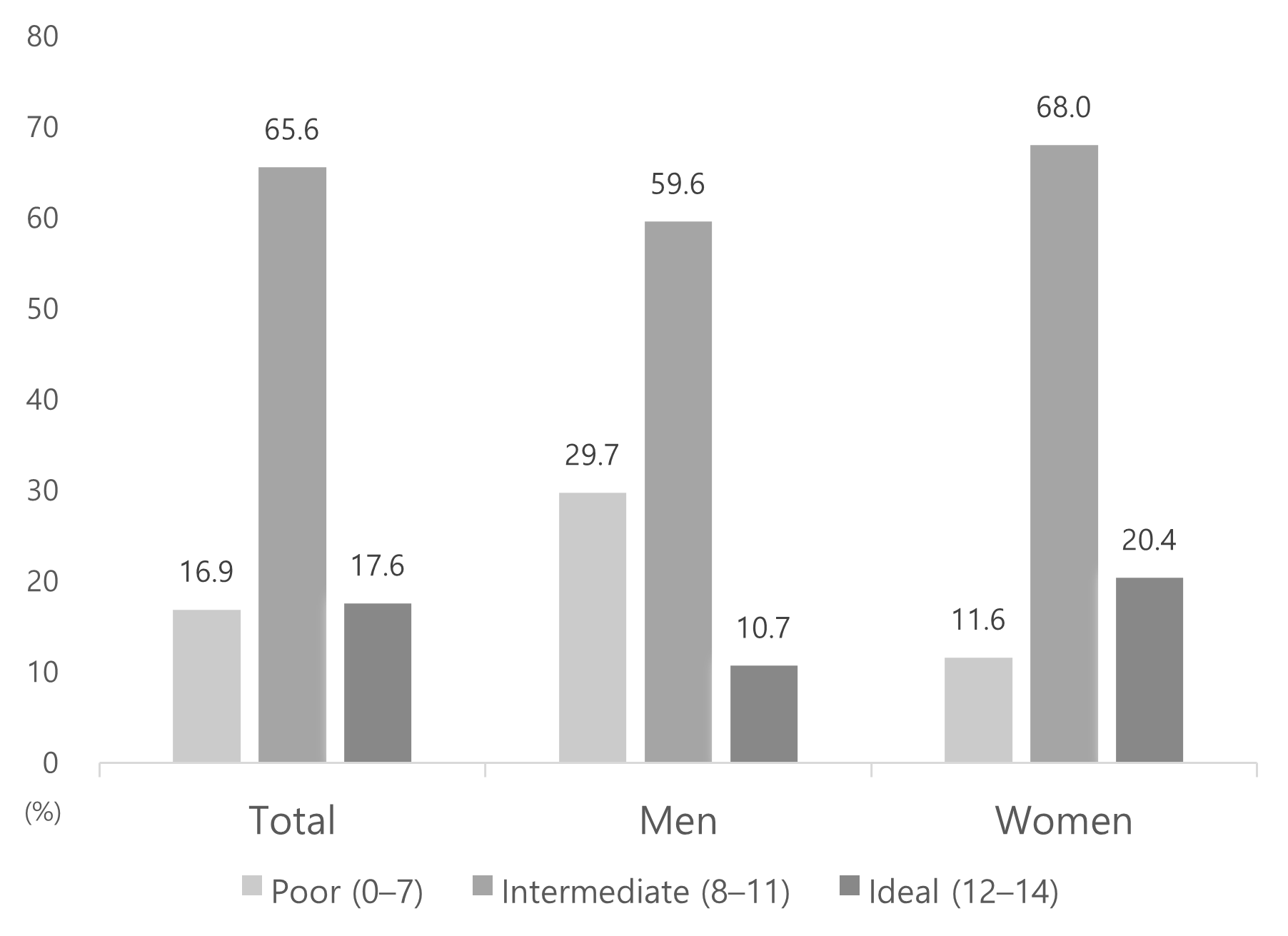


**Fig. S2.** Distribution of cardiovascular health metrics.

| **Table S1.** Components and cut-points in the Diet Quality Index for Koreans (DQI-K) | | |
| --- | --- | --- |
| Component | Score | |
|  | 0 | 1 |
| Daily protein intake (%)^a)^ | ≤100 | 100–150 (> 150 is given 2 points) |
| Percentage of energy obtained from fat (%) | <22.5 | ≥22.5 |
| Percentage of energy obtained from saturated fat (%) | <7 | ≥7 |
| Daily cholesterol intake (mg) | <300 | ≥300 |
| Daily whole-grain intake | Daily | Not daily |
| Daily vegetables intake (g) | ≥200 | <200 |
| Daily fruit intake (g) | ≥200 | <200 |
| Daily sodium intake (mg) | <2,000 | ≥ 2,000 |
| ^a)^ The daily recommended amount for those aged <65 years old is 60 g for men and 50 g for women. | | |

| **Table S2.** Definition of cardiovascular health metrics | | | |
| --- | --- | --- | --- |
| CVH metrics component | Poor (0 point) | Intermediate (1 point) | Ideal (2 points) |
| Smoking | Current smoker | Former smoker (≤12 mo) | Never smoked or quit smoking >12 mo ago |
| Physical activity | None | Moderate intensity for 1–149 min/wk or vigorous intensity for 1–74 min/wk or a combination of moderate and vigorous intensity for 1–149 min/wk | Moderate intensity ≥150 min/wk or vigorous intensity ≥75 min/wk or a combination both above ≥150 min/wk |
| Healthy diet (score)^a)^ | ≥6 | 3 to <6 | <3 |
| BMI (kg/m^2^)^b)^ | ≥25 | 23 to <25 | <23 |
| SBP/DBP (mmHg) | SBP ≥ 140 or DBP ≥ 90 | SBP 120 to <140 or DBP 80 to <90 or treated to goal | SBP <120 and DBP <80 and not treated for HTN |
| Fasting plasma glucose (mg/dL) | ≥126 | 100 to <126 or treated to goal | <100 and not treated for diabetes |
| Total serum cholesterol (mg/dL) | ≥240 | 200 to <240 or treated to goal | <200 and not treated |
| CVH, cardiovascular health; BMI, body mass index; SBP, systolic blood pressure; DBP, diastolic blood pressure.  ^a)^Diet scores were assessed using the diet quality index for Koreans (DQI-K). ^b)^BMI was categorized using validated values for Asian populations. | | | |

| **Table S3.** Baseline characteristics of participants and those excluded from the study | | | | |
| --- | --- | --- | --- | --- |
| Characteristic | Included people in the study (n = 2,928) |  | Excluded people in the study (n = 1,132) | P-value |
|  |  |  |  |  |
|  |  |  |  |  |
| Age (yr) | 51.8 ± 9.3 |  | 50.0 ± 9.3 | <0.001 |
| Men | 851 (29.1) |  | 575 (50.8) | <0.001 |
| Systolic blood pressure (mmHg) | 117.9 ± 14.9 |  | 120.5 ± 14.9 | <0.001 |
| Diastolic blood pressure (mmHg) | 75.5 ± 9.7 |  | 77.8 ± 10.2 | <0.001 |
| Body mass index (kg/m²) | 23.7 ± 3.0 |  | 24.2 ± 3.1 | <0.001 |
| Fasting glucose (mg/dL) | 88.0 (82–95) |  | 89.0 (83–97) | <0.001 |
| Total cholesterol (mg/dL) | 199.5 ± 35.5 |  | 195.6 ± 34.7 | 0.001 |
| Total Energy intakes (kcal/day) | 2,148.0 ± 758.2 |  | 2,927.0 ± 1,080.6 | <0.001 |
| Alanine aminotransferase (U/L) | 24.8 ± 17.2 |  | 27.5 ± 17.1 | <0.001 |
| Current smoker | 272 (9.3) |  | 284 (25.1) | <0.001 |
| Alcohol intake^a)^ | 369 (12.6) |  | 601 (53.1) | <0.001 |
| Hypertension | 711 (24.3) |  | 335 (29.6) | <.001 |
| Diabetes | 254 (8.7) |  | 107 (9.5) | 0.472 |
| Education level^b)^ | 1,395 (47.6) |  | 499 (44.1) | 0.086 |
| High income ($)^c)^ | 1,663 (56.8) |  | 612 (54.1) | 0.124 |

Data are presented as mean ± standard deviation, median (interquartile range), or number (%). The sum of the values for excluded people may not equal the total number of people in each group because some values were missing.

^a)^ ≥10 g of alcohol per day. ^b)^College or higher. ^c)^ Annual household income ≥$50,800.

| **Table S4.** Association between cardiovascular metrics categories and the risk of non-alcoholic fatty liver disease according to sex | | | | | | | | | | | | |  |
| --- | --- | --- | --- | --- | --- | --- | --- | --- | --- | --- | --- | --- | --- |
| Sex | CVH metrics category | No. of people | No. (%) of people with NAFLD^a)^ | | Unadjusted OR (95% CI) | | | | Adjusted OR (95% CI) | | | |  |
|  |  |  |  |  |  |  |  |  |  |  |  |  |  |
| Men | Poor | 253 | 201 | (79.5) | 3.91 | (2.75 | – | 5.56) | 2.25 | (1.38 | – | 3.66) |  |
|  | Intermediate | 507 | 252 | (49.7) | 1.00 |  |  |  | 1.00 |  |  |  |  |
|  | Ideal | 91 | 10 | (11.0) | 0.13 | (0.06 | – | 0.25) | 0.15 | (0.06 | – | 0.36) |  |
| Women | Poor | 241 | 143 | (59.3) | 6.47 | (4.84 | – | 8.65) | 3.43 | (2.35 | – | 4.99) |  |
|  | Intermediate | 1,413 | 260 | (18.4) | 1.00 |  |  |  | 1.00 |  |  |  |  |
|  | Ideal | 423 | 11 | (2.6) | 0.12 | (0.06 | – | 0.22) | 0.12 | (0.05 | – | 0.25) |  |

ORs (95% CI) adjusted for age, high-density lipoprotein cholesterol, triglycerides, HOMA-IR, alanine aminotransferase, income, education level and alcohol intake.

CVH, cardiovascular health; NAFLD, Non-alcoholic fatty liver disease; OR, odds ratio; CI, confidence interval.

^a)^NAFLD diagnosed using Fatty Liver Index.

| **Table S5.** Association between cardiovascular metrics and non-alcoholic fatty liver disease | | | | | | | | | | | | | |
| --- | --- | --- | --- | --- | --- | --- | --- | --- | --- | --- | --- | --- | --- |
| NAFLD diagnosis  method | Cardiovascular health metrics | No. of people | No. (%) of  people with  NAFLD | | Unadjusted  OR (95% CI) | | | | Adjusted  OR (95% CI) | | | | |
| **Biomarker-based** |  |  |  |  |  |  |  |  |  |  |  |  |  |
| NAFLD-LFS | **Category** |  |  |  |  |  |  |  |  |  |  |  |  |
|  | Poor | 494 | 268 | (54.3) | 5.67 | (4.58 | – | 7.02) | 2.63 | (1.82 | – | 3.80) |  |
|  | Intermediate | 1,920 | 332 | (17.3) | 1.00 |  |  |  | 1.00 |  |  |  |  |
|  | Ideal | 514 | 18 | (3.5) | 0.17 | (0.11 | – | 0.28) | 0.32 | (0.16 | – | 0.64) |  |
|  | **Continuous** |  |  |  |  |  |  |  |  |  |  |  |  |
|  | per 1.0  increase | 2,928 | 618 | (21.1) | 0.56 | (0.53 | – | 0.60) | 0.70 | (0.64 | – | 0.77) |  |
| HSI | **Category** |  |  |  |  |  |  |  |  |  |  |  |  |
|  | Poor | 494 | 283 | (57.3) | 6.51 | (5.25 | – | 8.07) | 4.56 | (3.43 | – | 6.06) |  |
|  | Intermediate | 1,920 | 328 | (17.1) | 1.00 |  |  |  | 1.00 |  |  |  |  |
|  | Ideal | 514 | 12 | (2.3) | 0.12 | (0.07 | – | 0.21) | 0.12 | (0.06 | – | 0.26) |  |
|  | **Continuous** |  |  |  |  |  |  |  |  |  |  |  |  |
|  | per 1.0  increase | 2,928 | 623 | (21.3) | 0.53 | (0.50 | – | 0.56) | 0.57 | (0.53 | – | 0.62) |  |
| **Both biomarker and image** | |  |  |  |  |  |  |  |  |  |  |  |  |
| Fatty liver index plus  LSR | **Category** |  |  |  |  |  |  |  |  |  |  |  |  |
|  | Poor | 494 | 163 | (33.0) | 5.20 | (4.07 | – | 6.65) | 1.97 | (1.44 | – | 2.70) |  |
|  | Intermediate | 1,920 | 166 | (8.7) | 1.00 |  |  |  | 1.00 |  |  |  |  |
|  | Ideal | 514 | 5 | (1.0) | 0.10 | (0.04 | – | 0.25) | 0.17 | (0.06 | – | 0.48) |  |
|  | **Continuous** |  |  |  |  |  |  |  |  |  |  |  |  |
|  | per 1.0  increase | 2,928 | 334 | (11.4) | 0.58 | (0.54 | – | 0.62) | 0.76 | (0.70 | – | 0.83) |  |
| NAFLD-LFS plus | **Category** |  |  |  |  |  |  |  |  |  |  |  |  |
| LSR | Poor | 494 | 141 | (28.5) | 4.93 | (3.80 | – | 6.38) | 1.68 | (1.18 | – | 2.39) |  |
|  | Intermediate | 1,920 | 144 | (7.5) | 1.00 |  |  |  | 1.00 |  |  |  |  |
|  | Ideal | 514 | 2 | (0.4) | 0.05 | (0.01 | – | 0.20) | 0.06 | (0.01 | – | 0.42) |  |
|  | **Continuous** |  |  |  |  |  |  |  |  |  |  |  |  |
|  | per 1.0  increase | 2,928 | 287 | (9.8) | 0.58 | (0.55 | – | 0.63) | 0.80 | (0.73 | – | 0.88) |  |
| HSI plus LSR | **Category** |  |  |  |  |  |  |  |  |  |  |  |  |
|  | Poor | 494 | 151 | (30.6) | 5.92 | (4.56 | – | 7.67) | 2.53 | (1.82 | – | 3.53) |  |
|  | Intermediate | 1,920 | 133 | (6.9) | 1.00 |  |  |  | 1.00 |  |  |  |  |
|  | Ideal | 514 | 2 | (0.4) | 0.05 | (0.01 | – | 0.21) | 0.05 | (0.01 | – | 0.36) |  |
|  | **Continuous** |  |  |  |  |  |  |  |  |  |  |  |  |
|  | per 1.0  increase | 2,928 | 286 | (9.8) | 0.54 | (0.51 | – | 0.58) | 0.68 | (0.62 | – | 0.74) |  |
| NAFLD, nonalcoholic fatty liver disease; OR, odds ratio; CI, confidence interval; LFS, Liver Fat Score; LSR, liver to spleen ratio; HIS, Hepatic Steatosis Index. | | | | | | | | | | | | | |
| Adjusted for age, sex, high-density lipoprotein cholesterol, triglycerides, HOMA-IR, alanine aminotransferase, income, education level and alcohol intake. | | | | | | | | | | | | | |

| **Table S6.** Association between the Cardiovascular Health in Ambulatory Care Research Team (CANHEART) health index, 10-year risk for atherosclerotic cardiovascular disease and the risk of non-alcoholic fatty liver disease | | | | | | | | | | | | | | | | | | | | | | | | |
| --- | --- | --- | --- | --- | --- | --- | --- | --- | --- | --- | --- | --- | --- | --- | --- | --- | --- | --- | --- | --- | --- | --- | --- | --- |
| Cardiovascular health metrics | No. of people | NAFLD by Fatty Liver Index | | | | | | | | | | |  | NAFLD by liver to spleen ratio | | | | | | | | | | |
|  |  | No. (%) of people with NAFLD | | Unadjusted  OR (95% CI) | | | |  | Adjusted  OR (95% CI) | | | |  | No (%) of people with NAFLD | | Unadjusted  OR (95% CI) | | | |  | Adjusted  OR (95% CI) | | | |
|  |  |  |  |  |  |  |  |  |  |  |  |  |  |  |  |  |  |  |  |  |  |  |  |  |
|  |  |  |  |  |  |  |  |  |  |  |  |  |  |  |  |  |  |  |  |  |  |  |  |  |
| **CANHEART index** |  |  |  |  |  |  |  |  |  |  |  |  |  |  |  |  |  |  |  |  |  |  |  |  |
| Category |  |  |  |  |  |  |  |  |  |  |  |  |  |  |  |  |  |  |  |  |  |  |  |  |
| Poor (0-3 score) | 493 | 286 | (58.0) | 3.29 | (2.68 | – | 4.03) |  | 1.60 | (1.19 | – | 2.15) |  | 165 | (33.5) | 2.93 | (2.34 | – | 3.67) |  | 1.46 | (1.11 | – | 1.93) |
| Intermediate (4–5 score) | 1,930 | 571 | (29.6) | 1.00 |  |  |  |  | 1.00 |  |  |  |  | 283 | (14.7) | 1.00 |  |  |  |  | 1.00 |  |  |  |
| Ideal  (6 score) | 505 | 20 | (4.0) | 0.10 | (0.06 | – | 0.16) |  | 0.06 | (0.03 | – | 0.12) |  | 25 | (5.0) | 0.30 | (0.20 | – | 0.46) |  | 0.47 | (0.30 | – | 0.75) |
| Continuous |  |  |  |  |  |  |  |  |  |  |  |  |  |  |  |  |  |  |  |  |  |  |  |  |
| per 1.0 higher | 2,928 | 877 | (30.0) | 0.23 | (0.20 | – | 0.27) |  | 0.36 | (0.28 | – | 0.45) |  | 473 | (16.2) | 0.33 | (0.28 | – | 0.40) |  | 0.61 | (0.49 | – | 0.76) |
| **10-year risk for ASCVD^a)^** |  |  |  |  |  |  |  |  |  |  |  |  |  |  |  |  |  |  |  |  |  |  |  |  |
| <5% | 1,631 | 364 | (22.3) | 1.00 |  |  |  |  | 1.00 |  |  |  |  | 197 | (12.1) | 1.00 |  |  |  |  | 1.00 |  |  |  |
| ≥5% | 816 | 379 | (46.5) | 3.02 | (2.52 | – | 3.62) |  | 1.54 | (1.10 | – | 2.15) |  | 192 | (23.5) | 2.24 | (1.80 | – | 2.79) |  | 1.38 | (0.97 | – | 1.96) |
| ASCVD, atherosclerotic cardiovascular disease; NAFLD, non-alcoholic fatty liver disease; OR, odds ratio; CI, confidence interval. | | | | | | | | | | | | | | | | | | | | | | | | |
| Adjusted for age, sex, high-density lipoprotein cholesterol, triglycerides, HOMA-IR, alanine aminotransferase, income, education level and alcohol intake. | | | | | | | | | | | | | | | | | | | | | | | | |
| ^a)^ Calculated among participants aged ≥40. | | | | | | | | | | | | | | | | | | | | | | | | |
